# Effect of Geographical Location on the Phenolic and Mineral Composition of Chetoui Olive Leaves

**DOI:** 10.3390/foods12132565

**Published:** 2023-06-30

**Authors:** Mariem Zakraoui, Hédia Hannachi, Igor Pasković, Nikolina Vidović, Marija Polić Pasković, Igor Palčić, Nikola Major, Smiljana Goreta Ban, Lamia Hamrouni

**Affiliations:** 1 Laboratory of Management and Valorization of Forest Resources, National Researches Institute of Water, Forests and Rural Engineering, University of Carthage, Ariana 2080, Tunisia; hamrounilam@yahoo.fr; 2 Faculty of Sciences of Tunis, University of El Manar, Tunis 2092, Tunisia; 3 Laboratory of Vegetable Productivity and Environmental Constraint, Department of Biology, Faculty of Sciences, University Tunis El Manar, Tunis 2029, Tunisia; hannachi_hedia@yahoo.fr; 4 Department of Agriculture and Nutrition, Institute of Agriculture and Tourism, Karla Huguesa 8, 52440 Poreč, Croatia; nikolina@iptpo.hr (N.V.); mpolic@iptpo.hr (M.P.P.); palcic@iptpo.hr (I.P.); nikola@iptpo.hr (N.M.); smilja@iptpo.hr (S.G.B.)

**Keywords:** *Olea europaea* L., phenols, minerals, environmental factors, Chetoui, oleuropein

## Abstract

In this study, we investigated the influence of pedological parameters and variation of altitude on the mineral nutrients, phenolic compounds, and antioxidant activities of olive leaves. Samples of the Chetoui cultivar were collected from eight geographical locations with different altitudes. Levels of phenolic compounds varied according to the altitude. Classification of the locations revealed that altitude 1 (>500 m) was characterized by high levels of secoiridoids and simple phenols, while altitude 2 (500–300 m) and altitude 3 (<300 m) were higher in flavonoids. Levels of Mn, Ca and B in the leaves and level of Zn in the soil were significantly correlated with the abundance of oleuropein and luteolin-7-*O* glucoside, the most important phenols in Chetoui olive leaves. The results suggest that, in addition to pedological criteria, environmental conditions also influence the formation of phenolic compounds.

## 1. Introduction

Olives (*Olea europaea* L.) are widely cultivated in Mediterranean countries. The olive trees produce olive oil and table olives. This crop is the main component of the cropping systems in most arid and semi-arid regions. The olive industry produces many olive by-products, among them olive leaves. The quantities of pruning waste were estimated at 25 kg of leaves and twigs per year and per tree [1,2].

Olive leaves are typically used in traditional medicine in the Mediterranean due to their therapeutic properties associated with their richness in polyphenols compounds. Numerous studies showed that the polyphenols from olive leaves have an antihypertensive effect [3,4], cardioprotective roles, neuroprotective characters [5], anti-inflammatory effects [6], and antimicrobial activities, with a possible anticancer impact [7,8]. In addition, olive leaves have found applications in animal feed. Several industries have increased their interest in natural plant extracts and by-products rich in bioactive compounds [9,10].

In Tunisia, more than 30% of cultivable lands are devoted to olive growing. The Chetoui olive is the secondmost common cultivated variety. Numerous studies showed that high contents of total phenols, tocopherols, and flavonoids, the secondary active compounds of the olive leaves, characterize the Chetoui variety [11,12,13]. Hence, the richness of bioactive compounds makes Chetoui an economically attractive olive variety.

In recent years, several industries have increased their interest in natural plant extracts and by-products rich in bioactive compounds [9]. The quantification of bioactive compounds is an important parameter to characterize and evaluate the olive and its impact on human health [14]. Oleuropein and its derivatives are the most important phenolic compounds in olive leaves [10]. These compounds have a major interest in the industrial sector, always seeking natural molecules to replace synthetic antioxidants. The concentration of polyphenols in olive leaves varies according to several factors, including genetic factors (variety), environmental factors (location), and cultural practices.

It has been noted that geographical location impacts phenol composition in olive leaves [14,15,16]. The response to pedoclimatic variations differs according to the variety [17]. However, despite the research on olive leaves, particularly those of the Chetoui variety, there are no studies about the pedo-climatic effect on the variation of mineral nutrients and phenolic compounds from olive leaves of the Chetoui Tunisian variety.

For this purpose, this study investigated the metabolic rearrangement of minerals and phenols contents, phenolic acids and flavonoids, and antioxidant capacity of olive leaves according to localities at different bioclimatic zones. The Chetoui is the second most common Tunisian autochthonous olive variety and is widespread in northern Tunisian orchards. The aim of this study is to (i) characterize and compare the minerals and phenolic compounds of each location, (ii) characterize the olive trees groves soils, and (iii) elucidate the eventual correlations between soil parameters and olive leaves biochemical compounds that can be used to determine the suitable pedological conditions for farming of olive trees for the production of olive leaves with richer active ingredients.

## 2. Materials and Methods

### 2.1. Plant Material

Olive leaves (*Olea europaea* L.) of the Chetoui variety were collected from different locations in the north of Tunisia (Table S1). Olive trees used in this study were equivalents in age, size, and other culture conditions. From each region, three trees were randomly collected at the postharvest fruits stage. Leaves were collected from the central part of olive shoots in a unified way around the tree [18].

Samples were dried at 50 °C with air circulation until constant weight, then were crushed using a laboratory blender. Methanol (MeOH) extracts were prepared as described previously [18].

### 2.2. Chemicals

The standards of phenolic compounds (analytical grade purity) were obtained from Extrasynthese (Genay, France). Methanol (MeOH) and acetonitrile (AcN) (HPLC grade purity) were obtained from Merck (Darmstadt, Germany), and phosphoric (HPLC grade purity) acid was obtained from Sigma-Aldrich (St. Louis, MO, USA), while hydrochloric acid (Suprapure) was procured from Merck (Darmstadt, Germany). Deionized water (HPLC grade purity) was procured by Siemens UltraClear (Siemens AG, München, Germany). The multi-element standard solution was obtained from Perkin Elmer (NexION Setup Solution, Waltham, MA, USA). Argon used to form plasma was supplied by Messer (Mes-ser Croatia Plin d.o.o., Zaprešić, Croatia) for the inductively coupled plasma mass spectrometric analysis (ICP-MS) was of purity 6.0 and, together with acetylene.

### 2.3. Measurement of the Total Antioxidant Capacity

Determination of antioxidant activity was carried out through a UV–visible spectrophotometer (Model UV-1800, Shimadzu Corporation, Kyoto, Japan) to estimate the 2,2-diphenyl-1-picrylhydrazyl (DPPH) free radical scavenging test [19] and by the ferric reducing ability of the plasma assay (FRAP) [20]. To determine the radical scavenging ability (DPPH), a mixture of 1 mL of leaf extract and 2 mL of 0.1 mM DPPH radical was prepared. Then, the mixture was incubated for 30 min in the dark at room temperature. The absorbance was measured at 517 nm. The results were assessed as mmol of Trolox equivalents/g dry weight (DW). As for the FRAP assay, a mixture of 1 mL of olive leaf extract with 2 mL of FRAP reagent was prepared, incubated for 4 min, and the absorbance was measured at 593 nm. Results were expressed as mmol of Fe^2+^ equivalents/g DW.

### 2.4. High-Performance Liquid Chromatography (HPLC)

HPLC analyses were performed according to the Major et al. (2022) method [21]. The HPLC instrument consisted of two solvent delivery units (Shimadzu Nexera LC-40DX3, Kyoto, Japan), an autosampler (Shimadzu Nexera SIL-40CX3, Kyoto Japan), a thermostated column compartment (Shimadzu Nexera CTO-40C, Kyoto, Japan), and a photo diode array detector (Shimadzu Nexera SPD-M40, Kyoto, Japan). The reversed-phase separation of the targeted compounds was achieved by injecting 5 µL of the sample on a C18, 2.1 mm × 150 mm, 2.7 µm core-shell column (Agilent, Palo Alto, CA, USA) held at 30 °C and using a binary gradient elution of mobile phase A (water/0.1% formic acid) and mobile phase B (acetonitrile/0.1% formic acid) at 0.35 mL/min: 0 min to 2 min: 95% A; 2 min to 20 min: 95% A to 50% A; 20 min to 21 min: 50% A to 5% A; 21 min to 23 min: 5% A; 23 min to 24 min: 5% A to 95% A; 24 min to 30 min: 95% A. Phenolic compounds in the extract were identified and quantified by comparing the retention time and peak area of analytical standards, respectively.

The UV/Vis detection was set at 360 nm for luteolin-4-*O*-glucoside, luteolin-7-*O*-glucoside, apigenin-7-*O*-glucoside, apigenin, luteolin and rutin, and at 280 nm for oleuropein, oleuropein aglycone, oleacein, catechin, tyrosol, hydroxytyrosol, and verbascoside, and at 210 nm for oleanolic acid, respectively (Figure S1). Identification was performed by comparing the retention times of the target compounds in the sample extracts with the retention times of pure standards. Quantification was conducted using the external standard method. The calibration curves for individual polyphenols were obtained by serial dilutions of the corresponding stock standard solutions: rutin (y = 0.2543x − 0.1389, coefficient of determination, R^2^ = 0.9999, recovery: 98.7 ± 0.58%), luteolin-7-*O*-glucoside (y = 0.4183x + 0.8289, coefficient of determination, R^2^ = 0.9990, recovery: 119.0 ± 2.9%), apigenin-7-*O*-glucoside (y = 0.2585x + 0.0679, coefficient of determination, R^2^ = 0.9991, recovery: 94.3 ± 3.1%), apigenin (y = 2.0227x + 1.5957, coefficient of determination, R^2^ = 0.9991, recovery: 86.7 ± 5.8%), luteolin (y = 1.3591x + 0.11185, coefficient of determination, R^2^ = 0.9993, recovery: 97.7 ± 0.6%), oleuropein (y = 0.0926x + 0.3361, coefficient of determination, R^2^ = 0.9993, recovery: 106.0 ± 0.0%), oleuropein aglycone (y = 0.3141x + 0.0072, coefficient of determination, R2 = 0.9998), oleacein (y = 0.3196x + 2.1937, coefficient of determination, R^2^ = 0.9991, recovery: 98.0 ± 3.0%), catechin (y = 0.2073x − 0.0272, coefficient of determination, R^2^ = 0.9996), tyrosol (y = 0.6757x − 0.66, coefficient of determination, R^2^ = 0.9990, recovery: 94.3 ± 3.1%), hydroxytyrosol y = 0.1824x + 0.0352, coefficient of determination, R^2^ = 0.9997, recovery: 89.2 ± 6.1%), verbascoside (y = 0.2604x − 0.158, coefficient of determination, R^2^ = 0.9993, recovery: 98.0 ± 3.0%) and oleanolic acid (y = 0.3439x + 0.7961, coefficient of determination, R^2^ = 0.9993).

### 2.5. Determination of Total Phenolic Content (TPC)

Total phenolic content was determined using the colorimetric Folin–Ciocalteu method [22] with some modifications. Briefly, 2 µL of the extract was mixed with 18 µL of distilled deionized (dd) water and 140 µL sodium carbonate (6%) was added. After 1 min, 140 µL of Folin–Ciocalteu reagent was added, and so obtained mixture was left to stand for 60 min at room temperature before the absorbance at 750nm was measured (Tecan Infinite 200 Pro M Nano +, Männedorf, Switzerland). The results were calculated against a standard curve of gallic acid (y = 3.7867x − 0.2144; serial dilutions of gallic acid (−12.5, 25, 50, 75, 100, 150, 250 mg/L; coefficient of determination, R^2^ = 0.9999, recovery: 102.0 ± 2.9%) and expressed as mg (Gallic acid equivalent) GAE/g DW.

### 2.6. Mineral Concentration Measurement

The analysis of minerals was determined using the method described by Vidović et al. [22]. In this experiment, 200 mg of dried olive leaf samples were accurately weighed into a microwave pressure vessel. After the addition of 6 mL of concentrated nitric acid and 2 mL of 30% hydrogen peroxide, samples were digested using a microwave system (Milestone ETHOS UP; Sorisole, Italy) over 40 min at 1800 W and 200 °C. After cooling, the solutions were diluted to 25 mL with deionized water and quantitatively transferred to the appropriate vials. One replicate per digestion method was created for each sample. Samples were then analyzed by an ICP-OES spectrometer (ICPE-9820; Shimadzu, Kyoto, Japan) equipped with an autosampler (AS-10; Shimadzu, Kyoto, Japan).

Multi-element (Inorganic Ventures, Christiansburg, VA, USA) standard solutions were used in order to control the plasma positioning and preparation of calibration standard solutions. The calibration standard was prepared by serial dilution of a stock solution (concentration range from 0.01 to 15 mg/L for microelements and from 0.125 to 100 mg/L for macroelements). The accuracy of a procedure was tested by certified reference materials (WEPAL, Wageningen, Netherlands) prepared in the same way as samples. The most suitable emission lines, i.e., those without background and spectral interferences, were selected for detection.

### 2.7. Soils Physico-Chemical Analysis

Soil samples were collected from olive orchards at a depth of 30–60 cm. Then, samples were air dried, sieved through a 2 mm diameter mesh, and stored until analysis. The physicochemical parameters of soil samples were analyzed according to international standards. Three replications were performed for each executed analysis.

The pH and electrical conductivity (EC) of each soil sample were determined according to Paredes et al. standard method [23]. Moreover, organic matter (OM) was evaluated by cold extraction of organic carbon and colorimetric determination at the 600 nm wavelength [24]. Phosphorus (P) available content was analyzed following the method cited by Magdich et al. [25]. The total organic carbon (TOC) content was assessed by the sulfochromic oxidation method (NF ISO 14235). Soil samples were characterized for (T CaCO_3_) contents using the volumetric method (NF ISO 10693). The soil saturation rate is the ratio of the sum of the exchangeable cations (Ca^2+^, Mg^2+^, K^+,^ and Na^+^) by the cation exchange capacity. The cation exchange capacity (CEC) and exchangeable bases of samples were determined using an NH_4_OAc (1 N, pH 7) method. Micronutrients Fe, Mn, and Zn were determined by the DTPA method.

### 2.8. Statistical Analysis

The results of experiments are expressed as the mean values ± S.E.M. (standard error of the mean) of three replicates. Samples were analyzed by one-way analysis of variance (ANOVA) using the SPSS software (SPSS version 8.0 for Windows) with location as the main factor, followed by Tukey multiple comparison test. The differences were considered to be significant when *p* < 0.05.

A data matrix comprising sample information (biological repeats for each sample) were generated and uploaded to MetaboAnalyst 5.0 “http://www.metaboanalyst.ca/ (accessed on 22 May 2023)” to perform analysis. Raw data were subjected to auto data scaling. Heatmap dendrogram and supervised partial least squares discriminant analysis (PLS-DA) were generated to examine the separation of data between different groups and to find the most useful variables among the investigated geographical location, which are grouped according to altitude levels: A1 (altitude of regions > 500 m), A2 (altitude of regions between 500–300 m), A3 (altitude of regions <300 m).

## 3. Results

### 3.1. Phenols and Antioxidant Activity

Results of antioxidant activity, total polyphenols content and phenolic compounds rate of Chetoui leaves from different locations are presented in Table 1, Table 2 and Table 3. The olive leaves from Bouarada 2 showed the highest TPC, followed by Nabeul 1 and Jendouba. The antioxidant activity obtained by the 2,2-diphenyl1-picrylhydrazyl (DPPH) and the ferric-reducing ability of the plasma (FRAP) assays showed that the highest values were founded in leaves of Beja 1 and the lowest one in leaves of Bouarada 3 (Table 1).

The phenols compounds obtained by HPLC analysis showed that, for simple phenols, the Bouarada 2 leaves displayed a high hydroxytyrosol concentration, followed by leaves of Nabeul and Jendouba, while the lowest concentration was found in Bouarada 3. Tyrosol was the most abundant compound in leaves from Beja 1 and the lowest in Bouarada 2 leaves.

The Bouarada 3 leaves showed the highest concentration of oleanic acid, while the lowest rate was noted in Bouarada 1. Verbascoside concentration was higher in Jendouba leaves than in Beja 2. Oleuropein was the major compound observed in all tested olive leaves. The leaves of Beja 1 and Zaghouan were characterized by the highest concentration, followed by Jendouba, Beja 2, Bouarada 2, and Nabeul leaves.

The Bouarada 1 was distinguished by the highest oleacein concentration compared to all the other studied locations. For ole-aglycone, Bouarada 3 and Zaghouan marked the highest concentrations, while Jendouba had the lowest ones.

The flavonoid profile showed seven compounds (Table 3). All studied locations exhibited a similar behavior of catechin. In contrast, Beja 2, Bouarada 2, and Zaghouan leaves contained the highest amount of rutin, while the lowest one was determined in Bouarada 3 leaves compared to all the other studied locations.

Bouarada 3 leaves contained the highest Luteolin-7-*O*-glucoside concentration, while the highest Luteolin-4-*O*-glucoside concentration was determined in Beja 1, Bouarada 2, Bouarada 3 and Zaghouan leaves. The lowest concentration of Luteolin-7-*O*-glucoside was found in leaves of Jendouba, Bouarada 1, Bouarada 2, and Nabeul, while Luteolin-4-*O*-glucoside was found in lower concentration in Bouarada 1. Regarding luteolin, the highest concentrations were noted in Bouarada 3 and Bouarada 1, followed by Beja 2 and Zaghouan leaves, and the leaves of Jendouba were characterized by the lowest concentration. As for apigenin-7-*O*-glucoside, the highest content was found in Jendouba olive leaves, while the Beja 1 and Zaghouan leaves had the lowest. The apigenin compound was detected at a high concentration in Bouarada 1 leaves and not in Jendouba, Bouarada 2, and Nabeul leaves.

### 3.2. Mineral Nutrients

The macro and micronutrient concentrations of olive leaves from different locations are presented in Table 4. The olive leaves from the Jendouba locality showed the highest amount of phosphorous (P), potassium (K), sulfur (S), zinc (Zn) and copper (Cu). However, the highest amounts of phosphorous (P), potassium (K), sulfur (S), zinc (Zn), and copper (Cu) were detected in olive leaves from the Jendouba locality. The Bouarada 3 leaves showed the highest amount of calcium (Ca), magnesium (Mg) manganese (Mn). The Nabeul olive leaves were characterized by a richness of iron (Fe) boron (B) silicon (Si). Finally, the Chetoui olive leaves from Bouarada 1 have the highest sodium (Na) concentration.

### 3.3. Physico-Chemical Analysis of Soil

The physicochemical analysis of soils from different studied locations is presented in Table 5. The analysis of the electrical conductivity shows that the soil salinity of the studied locations exhibited similar behavior. Beja 2 and Bouarada 1 contained higher pH concentrations compared to Jendouba; these soils are characterized by slightly alkaline soil.

The total limestone (T CaCO_3_) rate in the soil of Bouarada 1, Bouarada 2, Bouarada 3 and Beja 2 was significantly higher than those of Zaghouane and Beja 1, followed by the soils of Jendouba and Nabeul, which showed very low limestone rates. In the case of carbon (TOC), the highest contents were detected in the soils of Jendouba, Bouarada 1, and Zaghouan, while the lowest content was found in the soil of Nabeul. Regarding organic matter (OM), higher contents were found in Zaghouan soil than Beja 1 Soil. In-depth horizons and saturation percent exhibited similar behavior in the soil of all studied locations except in the soil of Nabeul, which had the lowest rate.

For mineral elements, the Bouarada 1 was the richest soil on phosphorus (P_2_O_5_), and the Beja 1 one Zinc (Zn). The highest iron (Fe) and manganese (Mn) concentrations characterized the Jendouba soil.

### 3.4. Classification of Olive Leaves

**Figure 1:**
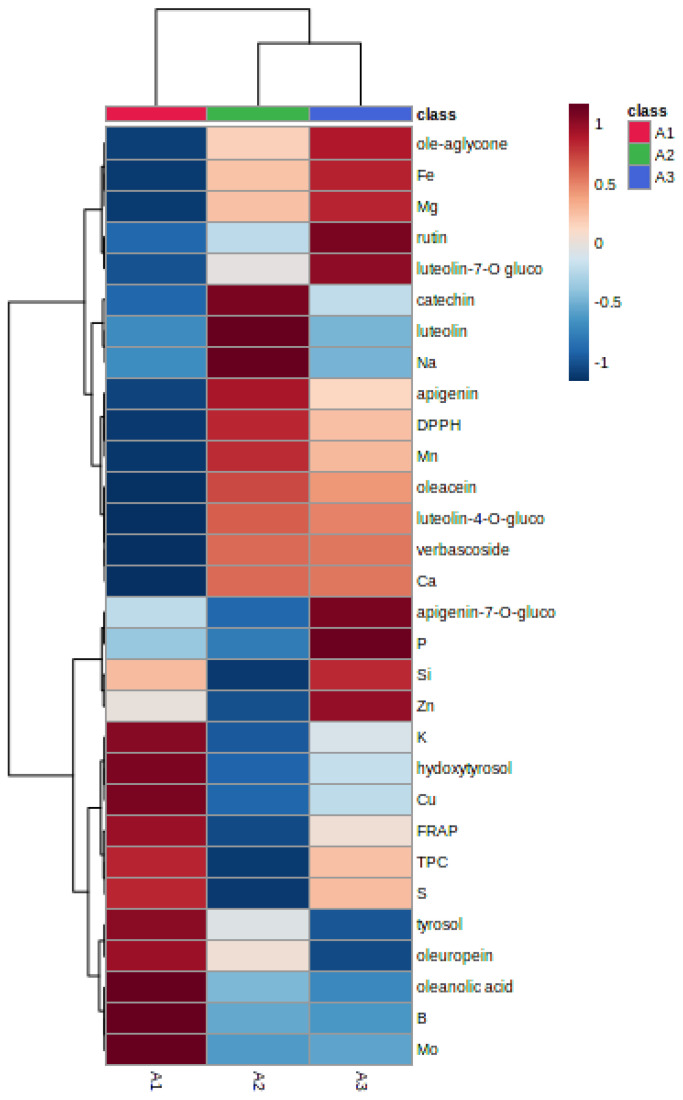
Heatmap along with HCA of olive leaves samples from different locations based on mineral nutrients, phenolic compounds and antioxidant activity. The rows in the heatmap diagram represent phenols/elements, and the columns represent samples. Sample codes were formed according to the altitude level: A1 (altitude of regions > 500 m), A2 (altitude of regions 500–300 m), and A3 (altitude of regions < 300 m).

In our study, an overall cluster analysis based on the determined metabolites, mineral nutrients, and antioxidant activity was performed to study the general differences among samples. Hierarchical clustering analysis (HCA) was conducted according to the altitude level of locations. Results showed a clear separation among the clusters established for different samples, as presented in the heatmap analysis (Figure 1). Applying Ward’s method based on Euclidean distance, olive leaf samples could be gathered into three different groups: group 1 (altitude > 500 m), group 2 (altitude 500–300 m), and group 3 (altitude < 300 m). Samples of group 1 were characterized by their richness in oleuropein, simple phenols, triterpenes, Mo, B, and K. While the 2 other groups were characterized by their richness in flavonoids and mineral nutrients.

**Figure 2:**
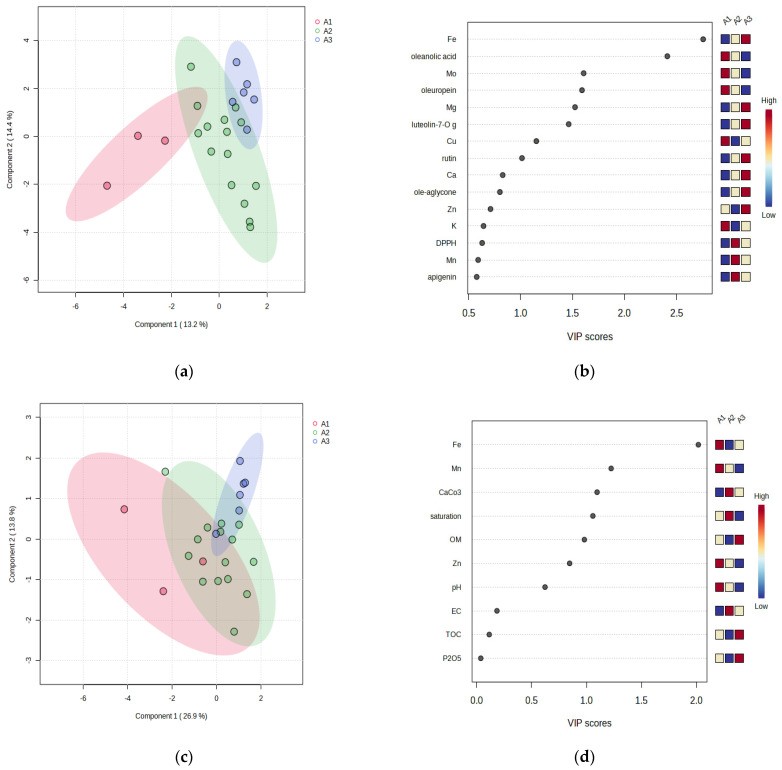
Partial least squares-discriminant analysis (PLS-DA) and the projection VIP scores of locations according to altitude. The PLS-DA is based on (**a**) the content of phenols and mineral nutrients in olive leaves and based on (**c**) the physicochemical parameters of soil. Associated VIP scores of the top parameters contribute to the variance of (**b**) phenols, minerals, and (**d**) and soil variance. The concentration of each parameter across the studied locations is shown by the color scale. The lowest ratios are in blue, and the highest ratio is in red.

To estimate the classification performance of the different olive leaves and soil characteristics, we conducted a supervised sparse version of the partial least-squares discriminant analysis (PLS-DA) among the different altitudes (Figure 2). PLS–DA differentiation showed the existence of three groups. However, for the soil, PLS-DA shows common parts between the three differentiated groups (Figure 2a,c). The contribution of mineral nutrients, phenols, and antioxidant activities variables to the discrimination between groups was evaluated through their VIP scores. A larger VIP score corresponds to a higher discriminating power. Oleuropein, Mo, Mg, and lutelin-7-O glucoside with VIP scores > 1.5 were selected as potential discriminants. Accordingly, Fe and oleanic acid were the most discriminant (VIP scores > 2) (Figure 2b). As for soil, Fe was the most discriminant parameter (VIP scores > 2), followed by Mn, CaCO_3_, saturation, and OM (VIP scores > 1) (Figure 2d).

## 4. Discussion

It is recognized that the compositions and concentrations of polyphenols in fruits and leaves vary according to several factors, such as the variety, the season, and the geographical location. The geographical location is one of the important factors of variation in olive leaves phenols composition. Commonly, high altitudes influence the concentrations of phenolic compounds [14,17,26]. The different locations investigated in this study are almost on the same bioclimatic floors but situated at different altitude levels.

In our study, the investigated locations showed a significant difference between phenolic compounds in olive leaves of the Chetoui variety. Previous studies reported that geographical location is among the factors that strongly influence phenolic composition [14,27].

Olive leaves are important for their richness in phenols. Among all, secoiridoids, in particular, oleuropein, is the major phenolic compound in leaves. The registered values of oleuropein differ significantly between the studied regions. This can be explained by the effect of pedoclimatic parameters. In fact, according to our study, a high concentration of Zn in soil positively correlates with oleuropein synthesis (*r =* 0.71, *p* < 0.05) (Table S2). Otherwise, in the class of simple phenols, a significant negative correlation was found between levels of hydroxytyrosol and levels of luteolin-7-*O* glucoside, luteolin, and apigenin. Considering the flavonoids, they present the second most abundant fraction after secoiridoids. The present results showed that luteolin-7-*O* glucoside, the most abundant compound in flavonoids, correlates negatively with apigenin-7-O glucoside. In addition, rutin correlates negatively with oleanic acids. For the pedological parameters, the results showed a significant positive correlation between CaCO_3_ and apigenin (*r =* 0.73, *p* < 0.05). Moreover, phenolic acids are a fraction with an important scavenging capacity. In this fraction, verbascoside was negatively correlated with CaCO_3_ (*r =* −0.78, *p* < 0.05). In addition, high pH rates in soil affect levels of verbascoside (*r =* −0.75, *p* < 0.05) (Table S2). In addition, Zhang et al. [16] showed that geographical location and soil fertility might influence phenolic compounds of virgin olive oil from the Leccino cultivar.

Our results showed that levels of TPC and FRAP were more important in the group of altitude 1 than in the other groups (Figure 1). Martínez-Navarro et al. [28] showed that the altitude factor was negligible, so it is probable that other factors had a greater effect on the higher content of the majority of TPC in the sampled leaves (Table 2). In another study conducted on Turkish olive cultivars, the leaves collected from different regions in Anatolia demonstrated that TPC decreased significantly when the geographical altitude decreased [15]. Taamalli et al. [14] observed that geographical location influenced the phenolic composition of TPC and antioxidant activity of Tunisian cultivars collected from different regions in Tunisia. In our results, a significant positive correlation was found between FRAP and oleuropein (*r =* 0.764, *p* < 0.05) and a significant positive correlation between DPPH and luteolin-4-O glucoside (*r =* 0.851, *p* < 0.01) (Table S5).

Concerning the direct correlation between pedological parameters and mineral nutrients in leaves, a significant negative correlation was detected between CaCO_3_ and K in leaves (*r =* −0.74, *p* < 0.05), and a significant positive correlation was detected between Fe and K in leaves (*r =* 0.78, *p* < 0.05). In addition, a significant negative correlation was found between pH and Cu, Mo, and S in leaves, i.e., alkaline soils affect the bioavailability of these nutrients in the leaves. Soil analysis also revealed that saturation correlates negatively with the bioavailability of B in leaves (*r =* −0.80, *p* < 0.05). As for Mn in soil, results showed that it has a significant positive correlation with Cu (*r =* 0.85, *p* < 0.01) and Zn (*r =* 0.74, *p* < 0.05) in leaves. It was also found that a high concentration of Zn in soil negatively correlates with levels of Ca (*r =* −0.78, *p* < 0.05), Fe (*r =* −0.75, *p* < 0.05) and Si (*r =* −0.72, *p* < 0.05) in leaves (Table S3). However, a recent study corroborated our results and showed that the elemental contents of the olive leaves were grouped depending on geographic area, and they can be used as a marker of the production area [29].

The mineral elements of plants are essential and intervene in polyphenol biosynthesis. For macronutrients in olive leaves, the higher accumulation of sodium (Na) concentration in leaves, especially in leaves of site Bouarada 1 (Figure 1, Table 3), reflects a possible contribution of a plant to mild salt stress. In this study, a significant negative correlation between the concentrations of Na and luteolin-4-glucoside (*r =* −0.77, *p* < 0.05) was found.

Phosphorus (P) is an essential mineral nutrient for growth. It plays an important role in energy storage and its transfer to the cells when they need it [27,30]. In our study, we observed a significant negative correlation between the concentrations of P in leaves and oleacein (*r =* −0.84, *p* < 0.01) (Table S4). Previous studies found that the concentration of P correlates negatively with the concentration of oleuropein and total phenols in olive leaves [31]. In contrast, Tekaya et al. (2016) showed a significant increase in the concentration of hydroxytyrosol and luteolin-7-O-glucoside to foliar application of fertilizers containing P [32]. This response due to P availability increases N accumulation, which is inversely proportional to the level of phenolic compounds due to competition between the synthesis of phenolic compounds and proteins in the same shikimic acid pathway Tekaya et al. (2016) [32]. It may be due also to higher P concentration which slowed down oleuropein synthesis, which resulted in a preserved concentration of its precursor, hydroxytyrosol [17].

In this study, a significant negative correlation between the concentrations of Calcium (Ca) and oleuropein (*r =* −0.79, *p* < 0.05) was determined (Table S4). The abundance of Ca in the leaves, particularly those of Bouarada 3, could be a method of adaptation of this local variety to calcareous soils [28]. Lukić et al. (2020) Observed a lower concentration of phenols in leaves related to a higher concentration of Ca in two studied sites [17]. Tekaya et al. (2016) observed a similar response of phenol concentrations to foliar application of fertilizers containing Ca [32]. Penel et al. (1999) proposed that Ca indirectly activates peroxydases, enzymes involved in the oxidative degradation of phenols, which results in a decrease in their concentration, as was the case in this work [33]. On the other hand, a significant negative correlation was found between Potassium (K) and Ca (*r =* −0.77, *p* < 0.05), which corroborates the results of Lukić et al. (2020) [17]. In contrast, a strong significant positive correlation was found between Magnesium (Mg) and Ca (*r =* 0.85, *p* < 0.01), which was previously reported by Stateras and Moustakas (2018) [34].

In the present results, among macronutrients in leaves, a significant positive correlation was found between Mg and oleanic acid (*r =* 0.72, *p* < 0.05) and between Mo and oleanic acid (*r =* 0.74, *p* < 0.05). Moreover, a positive correlation was revealed between Mn and luteolin-7-glucoside (*r =* −0.85, *p* < 0.01) and between Mn and ole-aglycone (*r =* 0.98, *p* < 0.01). In contrast, a strong negative correlation was found between Mn and apigenin-7-glucoside (*r =* −0.85, *p* < 0.01). On the other hand, a significant negative correlation between K and Mg was observed (*r =* −0.67, *p* < 0.05). This negative interaction was previously reported by Hartmann and Lilleland (1966) [35].

However, a significant negative correlation was revealed between boron (B) and apigenin-7-glucoside (*r =* 0.74, *p* < 0.05), while a significant positive correlation was revealed with hydroxytyrosol (*r =* 0.73, *p* < 0.05) (Table S4). Furthermore, previous studies showed that boron foliar application increase oleuropein content in olive leaves [36]. Moreover, a significant negative correlation was found between Zinc (Zn) and oleacein (*r =* −0.92, *p* < 0.001), and a significant positive correlation between Si and oleacein (*r =* 0.74, *p* < 0.05).

Results showed distinguished clusters are illustrated in Figure 1. As can be seen, the first group of leaves from regions located at altitude A1 more than 500 m are richer in secoiridoids, especially in oleuropein. Groups 2 (altitude A2) and 3 (altitude A3) presented the leaves richer in flavonoids. This may explain the negative correlation between secoiridoids and flavonoids (Table S6), which was confirmed by studies by Taamalli et al. (2018) [14]. On the other hand, Suleiman et al. (2020) showed a strong positive correlation between altitude and TPC (total phenolic content) and TFC (total flavonoid content) of Acacia honey. In contrast, they found that the TPC and TFC of Ziziphus honey were strongly negatively correlated with altitude [37]. It has also been revealed in recent research that the level of bioactive compounds of extra virgin olive oil strongly varied according to the altitude [38]. In addition, a study done on Mastoids olive cultivar showed that altitude influenced TPC, fatty acid, and phenol composition of fruit and oil [39].

In summary, slightly different soil characteristics could have had an indirect effect on the biosynthetic pathways of phenolic compounds. Indeed, mineral nutrients seem to depend mostly on edaphic characteristics and crop-growing techniques, while the water limitation inhibits the uptake of K, P, and other minerals, due to lower mobility in soils [30]. In addition, according to Martínez-Navarro et al. (2021), there are particular mineral nutrients, such as Mg and Fe, that could be useful for differentiating locations [28].

It has also been suggested that besides pedological parameters, many environmental factors (mean temperature, atmospheric pressure, rainfall, and radiation intensity) are related to the effect of altitude on the concentrations of phenolic compounds [38,40]. In addition, to be protected against oxidative stress, procured by severe weather conditions, olive trees improved phenolic compounds leaves, proving their important role in adaptation to severe climatic conditions. Other studies reported that the increase of polyphenol rates in harsh climatic conditions is an adaptation mechanism of plants to improve their resistance under high luminosity and temperature stress conditions [41,42].

## 5. Conclusions

In this study, the metabolic profile and mineral levels of olive leaves from different geographical locations were explored. The results of our study showed a significant variation in the behavior of phenolic compounds and mineral composition from different locations. Environmental factors, particularly geographical location and pedological factors may influence the nutritional potential of olive leaves. The studied soil data criteria showed that pH and CaCO_3_ are correlated with the levels of apigenin, verbascoside, and oleanic acid in leaves. In addition to pH and CaCO_3_, levels of Fe, Mn, and Zn in soil are correlated with variations of mineral contents in leaves. It is noted that the classification of the metabolites according to the altitude does not show an effect on the distribution of minerals in the leaves. The obtained results also revealed that the highest altitude (>500 m) is characterized by a high abundance of secoiridoids, mainly oleuropein. While the other two altitudes (<300 m) are more abundant in flavonoids. These variations may be useful for further research and give an insight into the biosynthetic pathway of phenolic compounds of the Chetoui variety in different climatic conditions. It would also be useful for future studies to evaluate and determine suitable environmental factors (rainfall, temperature, atmospheric pressure), which can be useful for farmers to produce higher levels of phenolics compounds in leaves.

## Figures and Tables

**Table 1:** Antioxidant activities DPPH (mmol/g DW TEQ) and FRAP (mmol/g DW Fe^2+^ EQ) and total phenols content (mg/100 g DW) in leaves collected from different locations.

Location	Antioxidant Activity		TPC
	FRAP	DPPH	
Jendouba	269.75 ± 5.04 a,b	256.26 ± 15.87 a,b	3968.33 ± 241.14 a,b
Beja 1	280.01 ± 4.99 a	273.88 ± 2.61 a	2940.33 ± 341.52 c,d
Beja 2	247.88 ± 23.56 a,b	243.37 ± 13.21 a,b	3485 ± 639.26 b,c
Bouarada 1	245.61 ± 20.94 a,b	236.15 ± 27.39 a,b	3313 ± 59.10 b,c
Bouarada 2	269.24 ± 31.13 a,b	270.28 ± 11.77 a,b	4308.33 ± 441.51 a
Bouarada 3	223.97 ± 15.45 b	266.72 ± 0.54 b	2378 ± 286.71 d
Zaghouan	263.65 ± 16.36 a,b	276.27 ± 3.57 a	3448.67 ± 219.51 b,c
Nabeul	260.64 ± 10.86 a,b	278.86 ± 2.67 a	4060.67 ± 83.06 a,b
*p*-value	*	**	***

Results are expressed as means ± standard errors (*n* = 3). Different lowercase letters in a column represent statistically significant differences between mean values for each main effect at *p* < 0.05 obtained by a one-way ANOVA and Tukey’s test. Mean values sharing the same letters are not statistically different. Significance at ***—*p* < 0.001, **—*p* < 0.01, *—*p* < 0.05.

**Table 2:** Concentrations of phenolic compounds (mg/100 g DW) in leaves collected from different locations.

Location	Triterpenes	Simple Phenols		Phenolics Acids	Secoiridoids		
	Oleanolic Acid	Tyrosol	Hydroxytyrosol	Verbascoside	Oleuropein	Oleacein	Ole-Aglycone
Jendouba	198.28 ± 14.41 b	22.6 ± 5.80 b,c	24.27 ± 2.16 b	58.5 ± 4.01 a	8604.27 ± 319.94 a,b	158.95 ± 3.50 f	18.33 ± 2.17 c
Beja 1	158.08 ± 7.01 b,c	34.85 ± 2.25 a	5.38 ± 0.75 d,e	40.92 ± 1.74 b,c	8998.17 ± 428.63 a	260.43 ± 10.10 c,d	26.53 ± 3.92 b
Beja 2	144.13 ± 37.98 b,c	21.32 ± 3.14 b,c	9.07 ± 1.60 c,d	23.72 ± 2.79 d	8152.73 ± 286.82 a,b	226.92 ± 4.47 d,e	21.62 ± 1.76 b,c
Bouarada 1	118.93 ± 24.55 c	29.6 ± 2.01 a,b	9.88 ± 2.01 c,d	31.92 ± 3.48 c,d	7141.4 ± 200.40 c	380.9 ± 18.48 a	27.88 ± 1.72 b
Bouarada 2	172.7 ± 10.59 b,c	19.87 ± 0.88 c	32.02 ± 3.11 a	27.32 ± 4.19 d	7801.34 ± 581.64 b,c	323.38 ± 28.46 b	21.72 ± 4,23 b,c
Bouarada 3	275.07 ± 10.65 a	23.95 ± 4.37 b,c	0.13 ± 0.23 e	49.85 ± 5.33 a,b	7120.48 ± 173.17 c	237.32 ± 7.80 c,d	36 ± 0.31 a
Zaghouan	141.12 ± 22.84 b,c	21.43 ± 1.71 b,c	13.42 ± 2.17 c	45.07 ± 1.02 b	8988.98 ± 407.75 a	195.25 ± 15.94 e,f	36.9 ± 3.99 a
Nabeul	175.33 ± 25.90 b,c	25.55 ± 1.72 b,c	25.52 ± 2.72 b	58.02 ± 4.60 a	7760.5 ± 199.28 b,c	273.67 ± 7.43 c	21.73 ± 2.38 b,c
*p*-value	***	***	***	***	***	***	***
RT (min)	29.64	8.49	13.63	11.86	14.75	15.25	20.56

Results are expressed as means ± standard errors (*n* = 3). Different lowercase letters in a column represent statistically significant differences between mean values for each main effect at *p* < 0.05 obtained by a one-way ANOVA and Tukey’s test. Mean values sharing the same letters are not statistically different. Significance at ***—*p* < 0.001.

**Table 3:** Concentrations of flavonoids (mg/100 g DW) in leaves collected from different locations.

Source of Variation	Flavonoids						
	Catechin	Rutin	Luteolin-7-O-Glucoside	Apigenin-7-O-Gluccoside	Luteolin	Luteolin-4-O-Glucoside	Apigenin
Jendouba	36.18 ± 4.93 d	237.47 ± 23.67 c	771.18 ± 38.91 c	76.92 ± 9.57 a	5.58 ± 0.62 d	34.75 ± 2.17 a,b	0 ± 0 e
Beja 1	41.3 ± 4.82 b,c,d	328.25 ± 19.84 b	1210.22 ± 116.93 b	36.72 ± 4.50 e	16.7 ± 0.87 b	39.37 ± 4.82 a	0.96 ± 0.24 d
Beja 2	50.8 ± 5.01 a,b	370.85 ± 10.83 b	1095,58 ± 35.92 b	45.62 ± 2.36 d,e	14.33 ± 0.97 b,c	27.25 ± 4.15 b,c	2.35 ± 0.17 c
Bouarada 1	44.83 ± 4.83 a,b,c,d	240.67 ± 32.69 c	769.68 ± 26.34 c	50.28 ± 4.43 c,d	24.18 ± 1.36 a	23.33 ± 2.65 c	3.88 ± 0.35 a
Bouarada 2	56.17 ± 6.08 a	357.5 ± 41.73 c	899.6 ± 52.16 c	65.37 ± 3.71 a,b	11.23 ± 2.5 c	41.6 ± 3.38 a	0 ± 0 e
Bouarada 3	55.42 ± 2.97 a	117.43 ± 6.05 a	1424.6 ± 74.94 a	21.82 ± 1.33 f	27.46 ± 2.77 a	41.85 ± 2.96 a	3.25 ± 0.55 a,b
Zaghouan	38.25 ± 3.04 c,d	393.32 ± 15.90 b	1103 ± 24.14 b	36.57 ± 1.53 e	14.03 ± 1.29 b,c	40.52 ± 1.14 a	2.48 ± 0.40 b,c
Nabeul	49.25 ± 1.59 a,b,c	324.72 ± 9.27 c	811.68 ± 50.17 c	61.77 ± 2.95 b,c	11.93 ± 2.05 b,c	34.5 ± 4.85 a,b	0 ± 0 e
*p*-value	***	***	***	***	***	***	***
RT (min)	7.47	11.13	12.25	13.87	16.79	3.59	20.06

Results are expressed as means ± standard errors (*n* = 3). Lowercase letters in a column represent statistically significant differences between mean values for each main effect at *p* < 0.05 obtained by a one-way ANOVA and Tukey’s test. Mean values sharing the same letters are not statistically different. Significance at ***—*p* < 0.001.

**Table 4:** Concentrations of macro and micronutrients in leaves collected from different locations.

	Macronutrients (g/kg DW)					Micronutrients (mg/kg DW)							
Location	P	K	Ca	S	Na	Mg	Fe	Zn	Mn	Cu	B	Si	Mo
Jendouba	1.01 ± 0.01 a	12.2 ± 0.51 a	20.47 ± 1.38 e	2.2 ± 0.03 a	4.08 ± 0.19 d	0.92 ± 0.02 d	69.49 ± 4.47 c	21.25 ± 0.78 a	14.29 ± 0.52 d	7.6 ± 0.21 a	7.68 ± 0.19 a,b	96.42 ± 9.06 b,c	0.29 ± 0.04 a
Beja 1	0.64 ± 0.02 d,e	9.85 ± 0.43 b,c	15.45 ± 0.48 f	1.44 ± 0.06 e	2.23 ± 0.29 f	0.61 ± 0.01 f	62.74 ± 3.93 c	14.41 ± 0.51 c	31.63 ± 0.66 b	3.58 ± 0.23 b,c	7.53 ± 0.28 a,b	103.58 ± 8.64 b,c	0.15 ± 0.01 c
Beja 2	0.98 ± 0.15 a,b	9.66 ± 0.1 b,c	22.86 ± 0.47 d,e	1.84 ± 0.03 c,d	6.89 ± 0.04 b	0.86 ± 0.005 d,e	73.18 ± 1.26 c	16.79 ± 0.34 b	24.04 ± 0.14 c	4.57 ± 0.24 b	6.78 ± 2.07 b,c	80.91 ± 5.52 c	0.16 ± 0.005 c
Bouarada 1	0.62 ± 0.15 d,e	7.64 ± 0.05 d	26.61 ± 0.24 b	1.72 ± 0.04 d	8.38 ± 0.19 a	0.89 ± 0.0 d,e	102.08 ± 3.13 a,b	7.96 ± 0.20 d	28.83 ± 0.68 b	2.55 ± 0.09 c	7.66 ± 0.49 a,b	235.83 ± 5.57 a	0.17 ± 0.01 b,c
Bouarada 2	0.6 ± 0.04 e	9.36 ± 0.11 c	23.02 ± 0.88 c,d,e	1.73 ± 0.1 d	5.38 ± 0.07 c	0.8 ± 0.02 e	100.3 ± 4.48 a,b	14.8 ± 1.13 c	24.75 ± 0.12 c	4.22 ± 0.36 b,c	8.23 ± 0.8 a,b	199.25 ± 14.17 a	0.24 ± 0.03 a,b
Bouarada 3	0.73 ± 0.03 e	5.78 ± 0.22 e	35.46 ± 1.84 a	2.01 ± 0.09 b,c	2.35 ± 0.03 e,f	2.25 ± 0.10 a	83.21 ± 2.21 b,c	14.25 ± 0.91 c	50 ± 2.39 a	3.97 ± 1.52 b,c	4.98 ± 0.33 c	167.08 ± 5.63 a,b	0.27 ± 0.02 a
Zaghouan	0.93 ± 0.2 c	9.21 ± 0.13 c	23.54 ± 0.47 c,d	2.07 ± 0.06 a,b	4.09 ± 0.05 d	1.45 ± 0.01 b	80.65 ± 6.10 b,c	18.38 ± 0.60 b	51.13 ± 0.78 a	4.95 ± 0.53 b	6.43 ± 0.23 b,c	134.38 ± 10.05 a,b,c	0.19 ± 0.01 b,c
Nabeul	0.68 ± 0.2 c,d	10.29 ± 0.19 d	25.63 ± 0.41 b,c	1.92 ± 0.04 b,c	2.68 ± 0.07 e	1.1 ± 0.02 c	121.13 ± 11.23 a	13.45 ± 0.45 c	25.33 ± 0.57 c	4.38 ± 0.13 b	9.79 ± 0.51 a	238.75 ± 4.50 a	0.24 ± 0.02 a,b
*p*-value	***	***	***	***	***	***	***	***	***	***	***	***	***

Results are expressed as means ± standard errors (n = 3). Lowercase letters in a column represent statistically significant differences between mean values for each main effect at *p* < 0.05 obtained by a one-way ANOVA and Tukey’s test. Mean values sharing the same letters are not statistically different. Significance: n.s.—not significant, ***—*p* < 0.001.

**Table 5:** Physicochemical parameters of soil sampled from different locations.

Location	CaCO_3_	pH	OM	Saturation	TOC	P_2_O_5_	EC	Mn	Zn	Fe
Jendouba	4.93 ± 1 c	7.61 ± 7.60 b	1.06 ± 0.23 a,b	44 ± 5.29 a,b	0.93 ± 0.17 a	15.3 ± 0.06 b,c,d,e	1.07 ± 0.12 a	107.5 ± 0.1 a	7.34 ± 0.14 b	570.63 ± 0.33 a
Beja 1	23.33 ± 1.52 b	8.05 ± 8.04 a,b	0.76 ± 0.48 b	47 ± 4.35 a	0.42 ± 0.04 b,c	18.23 ± 0.03 a,b,c,d	0.85 ± 0.95 a	34.53 ± 0.20 c	10.25 ± 0.13 a	385.18 ± 0.46 b
Beja 2	61 ± 4.35 a	8.12 ± 8.11 a	0.93 ± 0.05 a,b	51.67 ± 2.88 a	0.62 ± 0.09 b	21.17 ± 8.07 a,b,c	0.98 ± 0.03 a	45.17 ± 0.20 b	4.26 ± 0.06 c	128.3 ± 0.33 d
Bouarada 1	65.67 ± 2.08 a	8.13 ± 8.13 a	1.22 ± 0.33 a,b	47.33 ± 5.13 a	0.98 ± 0.13 a	26.45 ± 0.36 a	1.02 ± 0.11 a	7.08 ± 5.25 f	0.29 ± 0.03 g	27.68 ± 0.24 h
Bouarada 2	57.33 ± 2.51 a	7.95 ± 7.95 a,b	0.97 ± 0.02 a,b	48.67 ± 2.51 a	0.6 ± 0.14 b	9.94 ± 0.52 d,e	1.03 ± 0.07 a	24.53 ± 0.20 d	4.06 ± 0.06 c	117.88 ± 0.82 e
Bouarada 3	60 ± 13.22 a	7.75 ± 7.75 a,b	0.82 ± 0.18 b	51 ± 6.55 a	0.58 ± 0.07 b,c	12.41 ± 0.40 c,d,e	1.03 ± 0.24 a	12.38 ± 0.17 e	1.62 ± 0.11 e	48.27 ± 0.06 g
Zaghouan	38 ± 5 b	7.87 ± 7.87 a,b	1.57 ± 0.09 a	48.33 ± 5.77 a	0.95 ± 0.03 a	21.93 ± 3.63 a,b	0.86 ± 0.18 a	19.43 ± 0.15 d	2.4 ± 0.45 d	77.49 ± 0.36 f
Nabeul	5.8 ± 0.36 c	7.93 ± 7.93 a,b	0.91 ± 0.12 a,b	31.67 ± 1.52 b	0.29 ± 0.06 c	8.67 ± 0.01 e	0.79 ± 0.6 a	3.07 ± 0.06 f	1.01 ± 0.13 f	185.98 ± 10.11 c
*p*-value	***	*	*	**	***	***	n.s	***	***	***

Results are expressed as means ± standard errors (*n* = 3). Lowercase letters in a column represent statistically significant differences between mean values for each main effect at *p* < 0.05 obtained by a one-way ANOVA and Tukey’s test. Mean values sharing the same letters are not statistically different. Significance: n.s.—not significant, ***—*p* < 0.001, **—*p* < 0.01, *—*p* < 0.05.

## Data Availability

The data presented in this study are available on request from the corresponding author.

## Supplementary Materials

The following supporting information can be downloaded at: https://www.mdpi.com/article/10.3390/foods12132565/s1, Table S1. Geographical coordinates of different locations; Table S2. Correlation matrix between phenolic compounds in olive leaves and physicochemical parameters of soil.; Table S3. Correlation matrix between minerals in olive leaves and physicochemical parameters of soil.; Table S4. Correlation matrix between minerals and phenolic compounds in olive leaves.; Table S5. Correlation matrix between phenolic compounds and antioxidant activities in olive leaves.; Table S6. Correlation matrix between classes of phenolic compounds in olive leaves.; Figure S1. HPLC chromatograms showing the dominance of major phenolic compound peaks in olive leaves aqueous extract.
