# Supplementary material for: Effect of Geographical Location on the Phenolic and Mineral Composition of Chetoui Olive Leaves
Source: Foods. 2023 Jun 30;12(13):2565. doi: 10.3390/foods12132565 (PMC10340353; doi:10.3390/foods12132565)
Supplement: Supplementary file 1 [file foods-12-02565-s001.zip › foods-2454212-supplementary.pdf]

Table S1. Geographical coordinates of different locations

Region	Coordinates	Localisation
Bouarada 1	36°16′24″ N 9°30′07″ E	North West
Bouarada 2	36°16′08″ N 9°30′16″ E	North West
Bouarada 3	36°16′47″ N 9°29′25″ E	North West
Jendouba	36°49′27″ N 8°55′02″ E	North West
Beja 1	36°45′22″ N 9°28′03″ E	North West
Beja 2	36°42′32″ N 9°30′22″ E	North West
Zaghouane	36°27′41″ N 10°09′33″ E	North West
Nabeul	36°57′02″ N 10°53′28″ E	North East

Table S2. Correlation matrix between phenolic compounds in olive leaves and physico−chemical parameters of soil

		Rutin	Lut−7−O glucoside	Api−7−O glucoside	Lut−4−O glucoside	Luteolin	Catechin	Apigenin	Tyrosol	Verb	Oleuropein	Oleacein	Ole aglycone	Oleanic ac	HydroxyT
$CaCO_3$	r	−0.124	0.325	−0.465	−0.218	0.650	0.572	**0.730 ***	−0.181	**−0.783 ***	−0.537	0.500	0.381	−0.072	−0.396
	p value	0.770	0.433	0.245	0.604	0.081	0.138	0.040	0.669	0.022	0.170	0.207	0.352	0.865	0.331
pH	r	0.458	−0.046	−0.211	−0.527	0.299	0.244	0.379	0.386	**−0.755 ***	−0.126	0.666	−0.053	−0.703	−0.225
	p value	0.254	0.914	0.615	0.179	0.472	0.560	0.354	0.344	0.030	0.767	0.072	0.900	0.052	0.593
OM	r	0.325	−0.282	0.037	−0.113	−0.114	−0.504	0.252	−0.322	−0.014	0.242	−0.098	0.394	−0.476	0.123
	p value	0.431	0.499	0.930	0.790	0.788	0.203	0.547	0.437	0.974	0.564	0.818	0.334	0.233	0.772
Saturation	r	−0.101	0.539	−0.489	0.054	0.387	0.150	0.546	−0.157	−0.629	0.006	−0.035	0.384	0.047	−0.511
	p value	0.811	0.168	0.219	0.899	0.344	0.723	0.161	0.711	0.095	0.989	0.934	0.348	0.911	0.196
TOC	r	−0.097	−0.278	0.119	−0.299	0.003	−0.507	0.411	−0.245	−0.106	0.070	−0.102	0.220	−0.278	−0.038
	p value	0.819	0.504	0.779	0.472	0.995	0.200	0.311	0.558	0.803	0.870	0.810	0.601	0.505	0.928
P_2O_5	r	0.138	−0.026	−0.281	−0.601	0.300	−0.472	0.705	0.285	−0.429	0.157	0.128	0.288	−0.635	−0.509
	p value	0.745	0.951	0.500	0.115	0.470	0.238	0.051	0.495	0.289	0.710	0.763	0.490	0.090	0.198

EC	r	−0.500	−0.093	0.220	−0.157	0.101	0.215	0.219	−0.367	−0.272	−0.405	0.057	−0.170	0.297	0.042
	p value	0.207	0.826	0.601	0.710	0.811	0.608	0.602	0.371	0.514	0.319	0.894	0.688	0.476	0.922
Mn	r	−0.045	−0.235	0.518	−0.035	−0.633	−0.506	−0.405	−0.195	0.224	0.481	−0.644	−0.526	0.104	0.231
	p value	0.915	0.575	0.188	0.935	0.092	0.200	0.320	0.643	0.593	0.228	0.085	0.181	0.807	0.582
Zn	r	0.191	0.145	0.136	0.272	−0.436	−0.409	−0.471	0.347	0.027	**0.714 ***	−0.401	−0.356	−0.013	−0.016
	p value	0.651	0.732	0.747	0.515	0.280	0.315	0.239	0.400	0.950	0.047	0.325	0.386	0.976	0.970
Fe	r	−0.015	−0.250	0.513	0.115	−0.647	−0.590	−0.635	0.211	0.472	0.586	−0.535	−0.563	0.101	0.262
	p value	0.972	0.551	0.193	0.786	0.083	0.124	0.091	0.616	0.238	0.127	0.171	0.146	0.812	0.530

Pearson correlation coefficients (r) are bolded only for significant values.
The correlation is significant at the level 00.05 *, at the level 00.01 **

Table S3. Correlation matrix between minerals in olive leaves and physico−chemical parameters of soil

		B	Ca	Cu	Fe	K	Mn	Mg	Mo	N	P	S	Si	Zn
$CaCO_3$	r	−0.533	0.462	−0.599	0.033	**−0.745 ***	0.349	0.229	−0.333	0.601	−0.225	−0.252	0.146	−0.469
	p value	0.174	0.250	0.117	0.939	0.034	0.396	0.585	0.421	0.115	0.592	0.548	0.730	0.241
pH	r	0.209	−0.227	**−0.768 ***	0.177	−0.204	−0.066	−0.488	**−0.879 ****	0.537	−0.403	**−0.771 ***	0.184	−0.649
	p value	0.620	0.589	0.026	0.676	0.629	0.877	0.220	0.004	0.170	0.322	0.025	0.662	0.082
OM	r	−0.105	0.002	0.132	0.077	0.045	0.321	0.080	−0.132	0.367	0.357	0.418	0.074	0.142
	p value	0.805	0.997	0.756	0.857	0.915	0.439	0.850	0.755	0.371	0.386	0.303	0.861	0.737
Saturation	r	**−0.803 ***	0.101	−0.153	−0.612	−0.443	0.345	0.172	−0.279	0.343	0.186	−0.130	−0.473	0.076
	p value	0.016	0.812	0.717	0.107	0.271	0.403	0.684	0.503	0.406	0.660	0.759	0.236	0.857
TOC	r	−0.344	0.036	0.254	−0.238	−0.001	0.072	0.040	0.014	0.558	0.429	0.424	−0.136	0.159
	p value	0.405	0.932	0.544	0.570	0.998	0.865	0.924	0.973	0.150	0.289	0.295	0.748	0.707
P_2O_5	r	−0.324	−0.183	−0.265	−0.357	−0.139	0.147	−0.205	−0.696	0.625	0.251	−0.165	−0.244	−0.208
	p value	0.434	0.665	0.525	0.386	0.743	0.728	0.626	0.055	0.097	0.549	0.697	0.561	0.620
EC	r	−0.379	0.280	0.224	−0.207	−0.170	−0.249	0.100	0.398	0.456	0.134	0.249	−0.110	0.079
	p value	0.354	0.502	0.593	0.623	0.688	0.551	0.814	0.329	0.256	0.752	0.552	0.795	0.852
Mn	r	−0.027	−0.465	**0.850 ****	−0.620	0.691	−0.562	−0.304	0.337	−0.018	0.665	0.382	−0.694	**0.749 ***

	p value	0.950	0.245	0.008	0.101	0.058	0.147	0.464	0.415	0.967	0.072	0.350	0.056	0.032
Zn	r	0.025	**−0.782 ***	0.367	**−0.751 ***	0.553	−0.345	−0.503	−0.133	−0.340	0.183	−0.325	**−0.720 ***	0.483
	p value	0.954	0.022	0.372	0.032	0.155	0.403	0.204	0.754	0.411	0.664	0.432	0.044	0.225
Fe	r	0.275	−0.644	0.704	−0.492	**0.783 ***	−0.580	−0.428	0.280	−0.366	0.345	0.112	−0.527	0.588
	p value	0.510	0.085	0.051	0.215	0.022	0.131	0.290	0.503	0.372	0.403	0.791	0.180	0.125

Pearson correlation coefficients (r) are bolded only for significant values.

The correlation is significant at the level 00.05 * , at the level 00.01 **

Table S4. Correlation matrix between minerals and phenolic compounds in olive leaves.

		B	Ca	Cu	Fe	K	Mg	Mn	Mo	Na	P	S	Si	Zn
Rutin	r	0.427	−0.654	0.021	0.018	0.510	−0.600	−0.166	−0.530	0.182	0.152	−0.259	−0.180	0.217
	p value	0.292	0.079	0.960	0.966	0.197	0.116	0.694	0.177	0.666	0.720	0.535	0.669	0.606
Lut−7−Oglucoside	r	**−0.780 ***	0.306	−0.237	−0.486	−0.581	0.596	**0.727 ***	−0.136	−0.471	0.030	−0.124	−0.388	0.087
	p value	0.022	0.462	0.572	0.222	0.131	0.119	0.041	0.747	0.239	0.944	0.770	0.342	0.837
Api−7−O glucoside	r	**0.740 ***	−0.400	0.534	0.287	**0.763 ***	−0.593	**−0.857 ****	0.384	0.241	0.106	0.215	0.135	0.247
	p value	0.036	0.326	0.173	0.490	0.028	0.121	0.007	0.348	0.566	0.802	0.609	0.750	0.555
Lut−4−O glucoside	r	−0.226	0.037	0.206	−0.167	−0.069	0.396	0.460	0.405	**−0.775 ***	−0.108	0.099	−0.120	0.405
	p value	0.591	0.931	0.624	0.692	0.871	0.332	0.251	0.319	0.024	0.800	0.815	0.777	0.319
Luteoline	r	−0.563	0.651	**−0.749 ***	0.079	**−0.945 ****	0.546	0.623	−0.245	0.089	−0.442	−0.274	0.318	−0.690
	p value	0.146	0.081	0.032	0.852	0.000	0.161	0.099	0.559	0.833	0.273	0.511	0.443	0.058
Apigenin	r	−0.631	0.515	−0.567	−0.080	**−0.761 ***	0.394	0.513	−0.421	0.470	−0.034	−0.081	0.091	−0.514
	p value	0.093	0.192	0.143	0.851	0.028	0.334	0.194	0.298	0.240	0.937	0.850	0.830	0.193
Catechin	r	−0.070	0.558	−0.456	0.440	−0.550	0.280	0.088	0.136	0.076	−0.479	−0.220	0.402	−0.403
	p value	0.870	0.151	0.256	0.276	0.158	0.502	0.836	0.747	0.858	0.230	0.600	0.324	0.322
Tyrosol	r	0.144	−0.317	−0.495	−0.144	−0.094	−0.292	−0.002	−0.484	−0.179	−0.489	−0.680	0.072	−0.514
	p value	0.733	0.445	0.212	0.734	0.824	0.482	0.997	0.224	0.672	0.219	0.064	0.865	0.192
Verbascoside	r	0.132	0.140	0.513	0.076	0.251	0.380	0.070	0.634	−0.705	0.189	0.561	0.060	0.342
	p value	0.755	0.742	0.194	0.858	0.548	0.353	0.869	0.092	0.051	0.655	0.148	0.888	0.407

Oleuropein	r	0.057	**−0.797 ***	0.478	−0.614	0.663	−0.389	−0.044	−0.261	−0.319	0.491	−0.010	−0.694	0.676
	p value	0.893	0.018	0.231	0.105	0.073	0.341	0.917	0.533	0.441	0.217	0.981	0.056	0.066
Oleacein	r	0.327	0.156	**−0.820 ***	0.601	−0.433	−0.260	−0.086	−0.342	0.488	**−0.844 ****	−0.638	**0.748 ***	**−0.922 ****
	p value	0.429	0.713	0.013	0.115	0.284	0.534	0.840	0.408	0.220	0.008	0.089	0.033	0.001
Ole_aglycone	r	−0.697	0.501	−0.380	−0.103	**−0.733 ***	**0.724 ***	**0.980 ****	−0.150	−0.214	−0.067	0.092	0.080	−0.174
	p value	0.055	0.206	0.353	0.808	0.038	0.042	0.000	0.723	0.612	0.874	0.828	0.852	0.680
Oleanic_acid	r	−0.429	0.584	0.260	−0.092	−0.331	**0.727 ***	0.271	**0.749 ***	−0.622	0.004	0.397	−0.041	0.236
	p value	0.288	0.129	0.534	0.829	0.423	0.041	0.517	0.033	0.099	0.992	0.330	0.922	0.574
Hydroxytyrosol	r	**0.739 ***	−0.284	0.426	0.476	0.626	−0.424	−0.586	0.432	0.071	−0.046	0.209	0.300	0.248
	p value	0.036	0.495	0.292	0.234	0.097	0.295	0.127	0.285	0.867	0.914	0.619	0.470	0.554

Pearson correlation coefficients (r) are bolded only for significant values.

The correlation is significant at the level 00.05 * , at the level 00.01 **

Table S5. Correlation matrix between phenolic compounds and antioxidant activities in olive leaves.

		Rutin	Lut–7–*O* glucoside	Api–7–*O* glucoside	Lut–4–*O* glucoside	Luteolin	Catechin	Apigenin	Tyrosol	Verb	Oleuropein	Oleacein	Ole aglycone	Oleanic ac	HydroxyT
FRAP	r	0.644	−0.395	0.515	0.219	**−0.747 ***	**−0.729 ***	−0.517	0.204	0.065	**0.764 ***	−0.137	−0.458	−0.458	0.534
	p value	0.085	0.333	0.192	0.602	0.033	0.040	0.189	0.627	0.879	0.027	0.747	0.253	0.254	0.172
DPPH	r	0.224	0.280	−0.117	**0.851 ****	−0.244	−0.589	0.001	−0.014	0.487	0.379	−0.279	0.229	0.315	0.244
	p value	0.593	0.501	0.783	0.007	0.560	0.124	0.998	0.973	0.221	0.354	0.504	0.585	0.447	0.560

Pearson correlation coefficients (r) are bolded only for significant values.

The correlation is significant at the level 00.05 * , at the level 00.01 **

Table S6. Correlation matrix between classes of phenolic compounds in olive leaves.

		Flavonoides	Simple phenols	Phenolic acids	Secoiroides	Triterpenes
Flavonoides	r	1	**−0.713 ***	−0.255	**−0.961 ****	0.445
	p value		0.047	0.542	0.000	0.269
Simple_phenols	r	**−0.713 ***	1	0.181	0.631	−0.195
	p value	0.047		0.668	0.094	0.644
Phenolic_acids	r	−0.255	0.181	1	0.024	0.544
	p value	0.542	0.668		0.954	0.163
Secoiroides	r	**−0.961 ****	0.631	0.024	1	−0.673
	p value	0.000	0.094	0.954		0.067
Triterpenes	r	0.445	−0.195	0.544	−0.673	1
	p value	0.269	0.644	0.163	0.067	

Pearson correlation coefficients (r) are bolded only for significant values.

The correlation is significant at the level 00.05 * , at the level 00.01 **

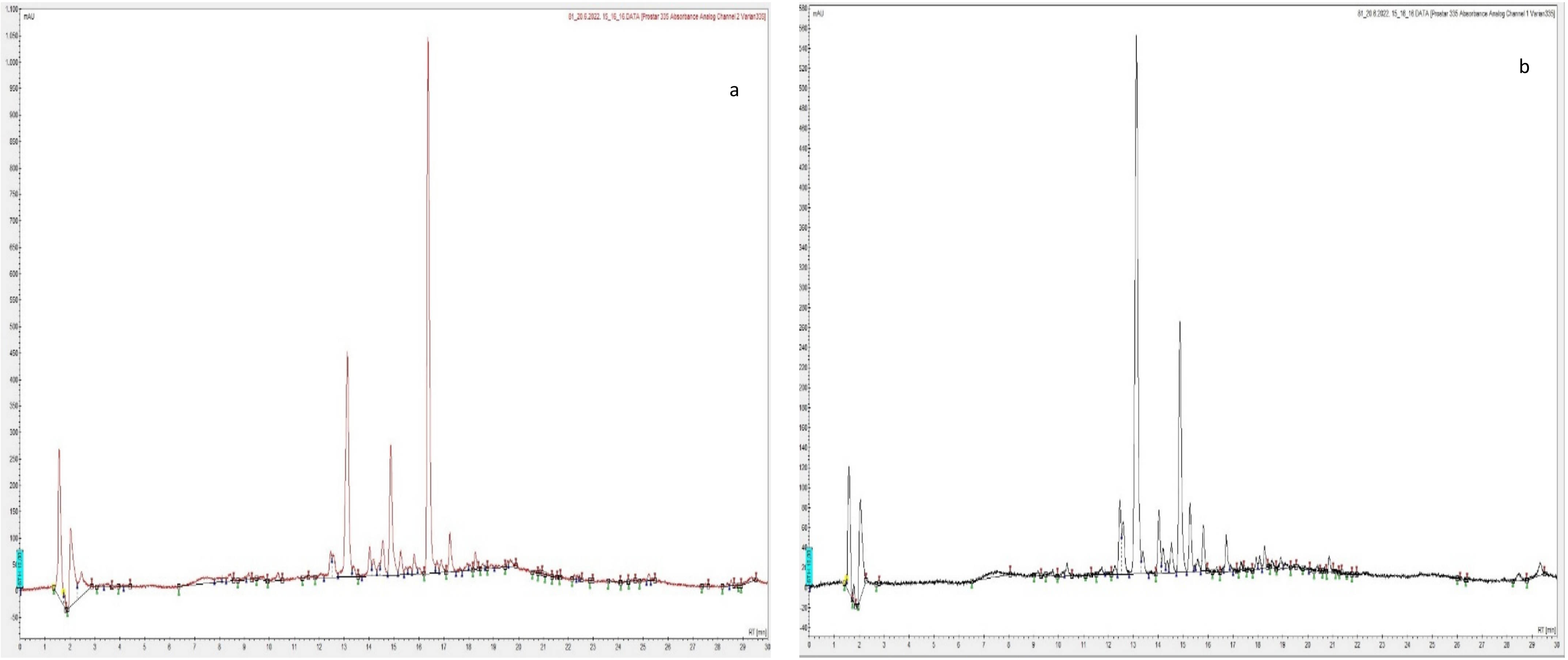

Figure S1. HPLC chromatograms showing the dominance of major phenolic compounds peak in olive leaves aqueous extract (a : Chromatogram at 280 nm, b : Chromatogram at 360 nm)

Acronyms :

Lut−4−O− glucoside : Luteolin−4−O−glucoside
Lut−7−O− glucoside : Luteolin−7−O−glucoside
Api−7−O− glucoside : Apigenin−7−O−glucoside
Verb : Verbascoside
Oleanic ac : Oleanic acid
HydroxyT : Hydroxytyrosol
Ole−aglycone: Oleuropein aglycone
TPC : Total Phenol Content
PRAP : ferric reducing ability of the plasma assay
DPPH : 20.2−diphenyl−1−picrylhydrazyl

T $CaCO_3$: T calcium carbonate
OM : Organic Matter
TOC : Total Organic Carbon
P_2O_5 : Phosphorus pentoxyde
CEC : Cation Exchange Capacity
EC : Electrical conductivity

Minerals : Boron (B), Calcium (Ca), Copper (Cu), Iron (Fe), Potassium (K), Magnesium (Mg), Manganese (Mn), Molybdenum (Mo), Sodium (Na), Phosphorus (P), Sulfur (S), Silicon (Si), Zinc (Zn).
